# The experiences of East Asian dementia caregivers in filial culture: a systematic review and meta-analysis

**DOI:** 10.3389/fpsyt.2023.1173755

**Published:** 2023-04-21

**Authors:** Qian Wang, Xiaohua Xiao, Jin Zhang, Dongdong Jiang, Amanda Wilson, Beiran Qian, Peige Song, Qian Yang

**Affiliations:** ^1^School of Public Health and the Department of Geriatrics, the Fourth Affiliated Hospital, Zhejiang University School of Medicine, Zhejiang University, Hangzhou, China; ^2^School of Applied Social Sciences, De Montfort University, The Gateway, Leicester, United Kingdom; ^3^Zhejiang University School of Medicine, Hangzhou, Zhejiang, China; ^4^School of Public Health and the Second Affiliated Hospital, Zhejiang University School of Medicine, Hangzhou, Zhejiang, China

**Keywords:** filial culture, dementia caregiver, systematic review, meta-analysis, East Asian

## Abstract

**Background:**

With the aging population in East Asia, the prevalence of dementia and the need for patient care is increasing. Family caregivers of people with dementia are at risk of physical and mental health problems. Filial piety culture regulates relationships within East Asian families and effects the well-being and behavior of dementia family caregivers (CGs).

**Objective:**

To systematically assess the experience of East Asian dementia caregivers in filial culture. Methods: Electronic databases, including MEDLINE, APA PsycINFO, CINAHL (*via* EBSCOhost), Web of Science, and Cochrane Library, were searched for relevant studies up to July 2021. Only original articles were included.

**Results:**

Thirteen eligible studies were included, of which eight were qualitative and five were quantitative. Meta-analysis showed a negative association (*r* = −0.18, 95%CI [−0.28, −0.08]) between filial culture and caregiver burden. The quantitative studies identified four themes related to dementia caregivers’ experiences: (1) Recognition and understanding of filial piety as part of cultural identity, (2) Role transitions- from child to CG, (3) Filial piety’s constraints on CGs; (4) CGs’ self-compassion through changing cultural norms of filial practice.

**Conclusion:**

Filial culture influences the whole process behind caregiving for East Asian dementia caregivers. At the same time, cultural transition has also brought about new connotations and practices to filial culture.

## 1. Introduction

Globally, population aging and longer life expectancy have led to a dramatic increase in the prevalence of dementia (1). In East Asia, the ratio of people 60 years and older is projected to increase from 13.8% in 2010 to 26% by 2030. The United Nations projects that this ratio will increase to 35.5% by 2050 (2). It is estimated that in 2019, 55.2 million people worldwide were living with dementia. The World Health Organization’s (WHO’s) Western Pacific Region (China, Japan, Singapore etc.) has the highest number of people with dementia (3). With this significant burden from dementia, there is a primary demand for care among this population (4).

People with dementia may experience memory loss, reduction in practical abilities, and changes in mood or behavior (5). These symptoms can oftentimes leave the patient dependent on a caregiver (CG), and the role of CG can be highly challenging (font-Variant, 2021). As a result, the well-being of CGs can be seriously impacted (6, 7). Most patients with dementia live at home and are cared for primarily by their children or other family members, who are the informal CGs. These informal CGs often provide years of extensive care for their relatives (8). They commonly lack professional knowledge and have limited care-related training (9). As a result, as proposed by Bertrand (10), informal CGs of older persons with dementia can face more challenges and experience higher levels of burden and depression than those caring for older persons without dementia. The term ‘caregiver burden’ has been widely used as an indicator of CGs’ experience in providing care to recipients (11). While the behavioral and psychological symptoms of dementia (BPSD) and the sociodemographic factors of the CG are the most significant factors affecting the burden of dementia, CGs’ socio-cultural influences are also important (12).

Culture is a complex construct (13) that influences people’s behavioral, cognitive, and affective processes (14, 15). Culture plays a crucial role in health behaviors, perception of illness, and even the etiology of dementia (16), all of which can contribute to delays in diagnosis and treatment as well as influencing the risk and resilience cycle that is part of dementia (17, 18). Equally, cultural factors influence CGs’ attitudes toward caregiving (19), including CGs’ appraisal of stress, coping strategies, and informal and formal support (20). Thus, there are cultural differences in the physical and mental health of dementia CGs (21). For example, compared with non-Latino white and Asian American caregivers, Latino and Black caregivers report more positive caregiving experiences and stronger cultural motivations for providing care (22). Cultural values like familism may have a negative effect on CGs’ health (23). One important aspect of East Asian culture is filial piety, which is rooted in Confucianism and has had a profound influence on East Asian societies (24, 25). It is believed that filial piety helps maintain social and family harmony (26) and can improve parental well-being. Older adults with filial children can get more support from relatives and friends (27). Chinese children’s filial beliefs affect their parents’ life satisfaction and loneliness (28).

Although industrialization and urbanization have weakened the bonds between people, filial piety is still highly influential in many East Asian communities. For instance, most Korean adult children still value and practice filial piety to care for their elderly parents (29). Furthermore, Lee and Sung (30) found that Korean caregivers expressed a significantly higher level of filial responsibility than American caregivers. In a filial piety framework, adult children are expected to provide financial, physical, and emotional support to care for their parents (31). And while this support can lead to CG challenges and burdens, filial piety can mediate these challenges. For example, research has shown that filial piety can indirectly affect the CG burden and serve as a protective function to reduce the harmful effects of stressors (32). It can also act as a protective factor against caregiver depression (33), and attitudes toward filial piety have been shown to be associated with CGs’ self-rated health status and overall well-being (34). Furthermore, filial piety can play an essential role in family care decision-making (35).

In the past few years, several meta-analyses and review articles have evaluated the effectiveness of interventions to support dementia CGs (8, 36–39). Although some interventions are culturally tailored, they are designed to target only language barriers or disease stigma. The development of culturally appropriate models for use with East Asian CGs requires a complete understanding of how core cultural values, such as filial piety, influence their appraisal and coping when caregiving (40). In addition, a systematic review described the impact of ethnicity and culture on Chinese-American CGs of dementia patients, suggesting that researchers should assess CGs’ adherence to filial piety (20). Understanding this cultural difference could help researchers to provide more targeted interventions for caregivers. However, few studies have examined the experience of dementia CGs in the context of filial culture in East Asia. This systematic review and meta-analysis therefore aimed to address this gap by identifying the experiences of filial cultural for East Asian dementia CGs.

## 2. Methods

### 2.1. Protocol and registration

The protocol was registered in the International Prospective Register of Systematic Reviews (CRD42021262529), and the systematic review was conducted following the Preferred Reporting Items for Systematic reviews and Meta-Analyses (PRISMA) guidelines (41).

### 2.2. Search strategy and selection criteria

A systematic search was conducted between 17^th^ of May 2021 and 30^th^ of June 2021 using the following electronic databases: MEDLINE, APA PsycINFO, CINAHL (*via* EBSCOhost), Web of Science, and Cochrane Library. To ensure literature saturation, we searched the reference lists from primary relevant articles and the “Related articles” option in MEDLINE. Keywords used included: “Alzheimer disease,” “Alzheimer’s disease,” “Dementia,” “vascular dementia,” “frontotemporal dementia” “FTD,” “Lewy body dementia,” “cognitive decline,” “filial piety,” “filial responsibility,” “filial obligation,” “filial duty,” “caregiver,” “family caregiver,” “informal caregiver,” “carer” and “nursing.”

This review used the SPIDER (Sample, Phenomenon of Interest, Design, Evaluation, Research type) model as a search strategy tool (42). Participants must be the CGs of a relative diagnosed with dementia or other cognitive impairments. We excluded studies that covered formal CGs because their relationship to the person with dementia and experience will differ from those of family CGs. Participants were East Asian or self-identified as East Asian (including Japanese, Korean, and Chinese). This review included articles of all study designs to examine the experiences of CGs in East Asia. Articles should evaluate the impact of filial culture on caregiver burden, cognition, and behavior. Quantitative, qualitative, and mixed-methods articles were included. Studies finished and published after the 1997s were included. Only original research articles were included. Reviews, commentaries, and editorials were excluded.

### 2.3. Study selection and data extraction

Literature search results were transferred to a reference management software (Zotero) and duplicates were deleted. Two authors (WQ, ZJ) screened titles and abstracts independently according to predefined inclusion and exclusion criteria. Then WQ and ZJ retrieved the full text of the studies and identified eligible studies. Disagreement between reviewers was resolved through discussion with a third reviewer (BR).

For all included studies, data was independently extracted into a predesigned form by two authors (WQ, ZJ). Extracted information included data source, study setting, design, sociodemographic characteristics of the CGs, and outcomes. The measures and results of filial piety and caregiver burden were retrieved from the quantitative studies. Themes related to carers’ experiences were extracted from qualitative studies.

### 2.4. Data analysis

A systematic review was conducted, and the corresponding information is presented in text and tables to summarize and explain the characteristics and results of the included studies. The findings of the qualitative and quantitative studies are reported separately to allow us to conduct a meta-analysis of the quantitative studies. In the meta-analysis, the variable of interest was the relationship between filial piety and caregiver burden. All effect sizes were converted to the Pearson correlation coefficient (*r*). For studies where correlation coefficients were not available, but standardized regression coefficients were present (*n* = 2), we used the imputation formula: r=β+0.05λ (*λ* = 1 for *β* ≥ 0, *λ* = 0 otherwise; all *|β|* < 0.5) (43). The analysis was conducted with a random-effects model (44). Using correlation coefficients to calculate Fisher’s Z 95% confidence interval (CI), we then transformed Fisher’s Z 95% CI to r 95% CI and examined the data by a forest plot. When assessing statistical heterogeneity, there is much uncertainty in measures such as *I^2^* when few studies (*n* = 4) are included (45). So, we assessed heterogeneity by using a chi-squared test with a significance level of *p* < 0.10 instead of the frequently used *p* < 0.05. Heterogeneity was assessed by *I^2^*, an *I^2^* ≥ 75% was classified as considerable heterogeneity; 40% < *I^2^* < 75% as moderate heterogeneity, and *I^2^* ≤ 40% as unimportant. The causes of heterogeneity were explored through sensitivity analysis. Analyses were conducted in Stata (Stata/SE 16.0) using the *maten* package.

The convergent integrated approach suggested by Joanna Briggs Institute (JBI) was used to integrate the findings of qualitative and qualitative studies (46). As codifying quantitative data is less error-prone than attributing numerical values to qualitative data, quantitative data was ‘qualitized’. The converted data was integrated by thematic synthesis through the following steps, coding the extracted data, grouping the codes, and then creating a specific theme (47).

### 2.5. Quality appraisal

Study quality was assessed by two reviewers (WQ, ZJ) with a quality appraisal tool, the Mixed Methods Appraisal Tool MMAT (48). The MMAT is a critical appraisal tool that is designed for the appraisal stage of systematic reviews. It was used to appraise the quality of the qualitative research and quantitative descriptive studies included. Each study was evaluated against the MMAT checklist, with every question responded to with one of three options “Yes,” “No,” or “Cannot tell.” Two reviewers (WQ, ZJ) were assigned to assess the selected articles independently. Studies with low methodological quality were not excluded as suggested by the MMAT. The studies had quality disagreements that were resolved through discussions with a third reviewer.

## 3. Results

### 3.1. Study selection

**Figure 1:**
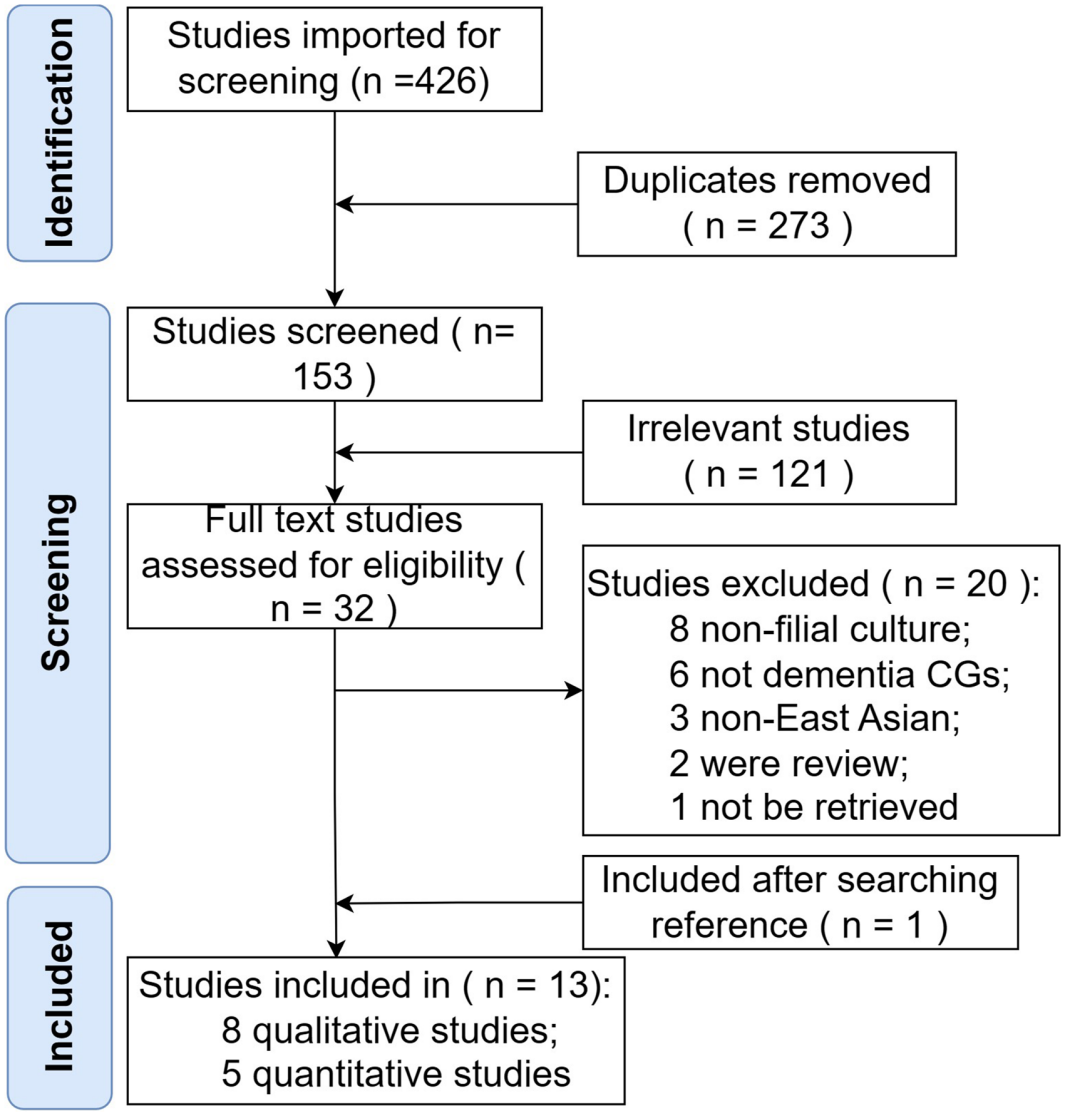
Flowchart of the search strategy.

The search strategy resulted in 426 records (Figure 1). After the removal of duplicates, 153 articles remained. The researchers screened titles and abstracts in the first round, removing protocols, studies of non-East Asian populations and non-dementia CGs, and studies that did not mention filial culture. The remaining 32 articles were further screened using the full texts, of which 20 were excluded for the following reasons: eight had no filial culture mentioned, six involved a non-dementia patient population, one was published in Korea and the full text was not available or made available to the team by the corresponding author, three did not include an East Asian population, and two were systematic reviews. One additional article was included after searching the reference lists of identified studies. Ultimately, thirteen studies than fulfilled the selection criteria and were included in the analysis. Of the fourteen, eight were qualitative studies and five were quantitative studies.

### 3.2. Study characteristics

The oldest study was published in 1997 in Korea, and most studies were (*n* = 8) published in the 2010s. The five quantitative studies had a total of 709 participants, of which three studies occurred in Koreans and two in Chinese. Two studies (30, 49) were published from recruitment of the same respondents. The key findings from the quantitative studies are listed in Table 1. Combined, the eight qualitative studies included in our study had 110 participants; six of them were in Chinese and two in Korean. Four studies (35, 51, 52, 56) resulted from the same two respondents. Characteristics, measures, and outcomes of the qualitative studies are listed in Table 2.

**Table 1:** Characteristics, measures, and outcomes of filial piety on dementia caregivers in qualitative studies.

Author(s) (Year)	Sample size (*n*)/Gender M/F (FP)/Ethnicity	Relationship with the patient	Data collection/Data analysis	Main result
Koo et al. (2021) (50)	*n* = 9, 5/4 (44%), Chinese	Children (78%) Other relatives^#^ (22%)	Semi-structured interviews/Narrative analysis	Three themes were identified: (1) family values, the cultural context of everyday care; (2) family support, everyday access to family and service networks; (3) family bonds, the maintenance family relations.
Zhang and Zhang (2020) (55)	*N* = 14, 5/9 (64%), Chinese	Spouse (36%) Children (57%) Other relatives^#^ (7%)	Semi-structured interview/IPA	Four key themes were found: (1) ‘being filial’; (2) ‘changing self and self-care’; (3) ‘seeking help’; (4) ‘having hope and continuing life’
Zhang et al. (2019) (35)	*N* = 14, 5/9 (64%), Chinese	Spouse (36%) Children (57%) Other relatives^#^ (7%)	Semi-structured interview/IPA	(1) Being filial is a cultural continuity and my future investment; (2) The changed perception and ways of being filial; (3) Filial responsibility is a social and cultural convention, but not my personal choice.
Chang et al. (2011) (51)	*N* = 30, 11/19 (63%), Chinese	Spouse (17%) Children (47%) Other relatives^#^ (36%)	In-depth semi- structured interview/Thematic Analysis	Factors influencing CGs’ decisional conflict: (1) Chinese value of filial piety; (2) limited financial resources and information; (3) placement willingness of the older adult; (4) family disagreement; (5) distrust of nursing home care quality; and (6) limited nursing home availability. Factors influencing CGs’ decisional conflict post-placement: (1) disappointment with nursing home care quality; and (2) self-blame for the placement decision.
Chang and Schneider (2010) (52)	*N* = 30, 11/19 (63%), Chinese	Spouse (17%) Children (47%) Other relatives^#^ (36%)	In-depth semi-structured interview/Systematic method	The traditional definition of filial piety was broadened to a multi-dimensional practice. Some regarded filial piety as a good nursing home and frequent family visits. While others believe nursing home placement is a non-filial behavior
Kim (2009) (53)	*N* = 8, 7/1 (88%), Korean	Spouse (50%) Children (38%) Other relatives^#^ (12%)	In-depth semi- structured interview/Transcendental Phenomenological Analysis	(1) Caring for loved ones demonstrates filial piety; (2) Adult children’s filial duty to their aging parents changed; (3) Parents believed they would not depend on their children and would not impose this expectation on their children.
Che et al. (2006) (54)	*N* = 9, 0/9 (100%), Chinese	Not tell	In-depth interview/Grounded Theory Method	The sense of “filial piety” and “feeling of out of control” are the two triggers that initiate the dementia CG role.
Chee and Levkoff (2001) (55)	*N* = 10, 0/10 (100%), Korean	Children (20%) Other relatives^#^ (80%)	In-depth interview/Thematic coding	Impact of filial culture on CGs: (1) delayed recognition of dementia symptoms; (2) is the primary motivation for providing care; (3) reluctant to seek assistance from outside of the home; (4) with a lack of legal services, filial sacrifice may result in intergenerational conflicts, CGs’ dissatisfaction, and family dysfunction. But filial responsibility is re-instituting.

CG, caregiver; CR, care receiver; IPA, Interpretative Phenomenological Analysis. ^#^Daughter-in-law included in other relatives.

**Table 2:** Characteristics, measures, and outcomes of filial piety on dementia caregivers in quantitative studies.

Author(s) (Year)	Study design sample size ethnicity	Caregiver age (M ± SD)/Gender, M/F (FP)	Relationship with the patient	Filial piety Measure/Caregiver burden measure	Correlation coefficient (*r*)
Lee et al. (2018) (57)	Cross-sectional study *N* = 98 Korean	51.77 ± 10.91 0/98 (100%)	Children (51%) Other relatives^#^ (49%)	Cicirelli’s (1991) 7item scale/Korean version of ZBI	*r* = 0.26
Yu et al. (2016) (85)	Cross-sectional study *N* = 401 Chinese	48.06 ± 8.49150/251 (63%)	Children (85%) Other relatives^#^ (15%)	RFPS/Chinese version of ZBI	*r* = −0.23
Chou et al. (1999) (86)	Cross-sectional study *N* = 150 Chinese	52.4 ± 14.7 35/115 (77%)	Spouse (30%) Children (32%) Other relatives^#^ (38%)	MS & CS/CBI	*r* = −0.10
Lee and Sung (1998) (30)	Cross-sectional study *N* = 60 Korean	44 ± 10 10/50 (83%)	Children (45%) Other relatives^#^ (55%)	Filial Expectancy Scale/BI & CBI	*r* = −0.15
Lee and Sung (1997) (49)	Cross-sectional study *N* = 60 Korean	44 ± 10 10/50 (83.3%)	Children (45%) Other relatives^#^ (55%)	Filial Expectancy Scale/BI & CBI	*r* = −0.15

ZBI, Zarit Burden Interview; RFPS, Reciprocal Filial Piety Scale; BI, Burden Interview; CBI, Caregiver Burden Inventory; MS, Montgomery obligation subscale; CS, Cicirelli’s obligation scale. ^#^Daughter-in-law included in other relatives.

### 3.3. Study quality

The quality appraisal tool of the MMAT discouraged calculating an overall score from the ratings of each criterion, so further details were provided for each criterion to inform the quality of the included studies. We evaluated all studies and found: (1) all qualitative studies met the criteria outlined by the MMAT; (2) all quantitative studies did not identify the risk of nonresponse bias, and one study did not specify the relationship between sampling strategy and study objectives.

### 3.4. Meta-analysis finding

**Figure 2:**
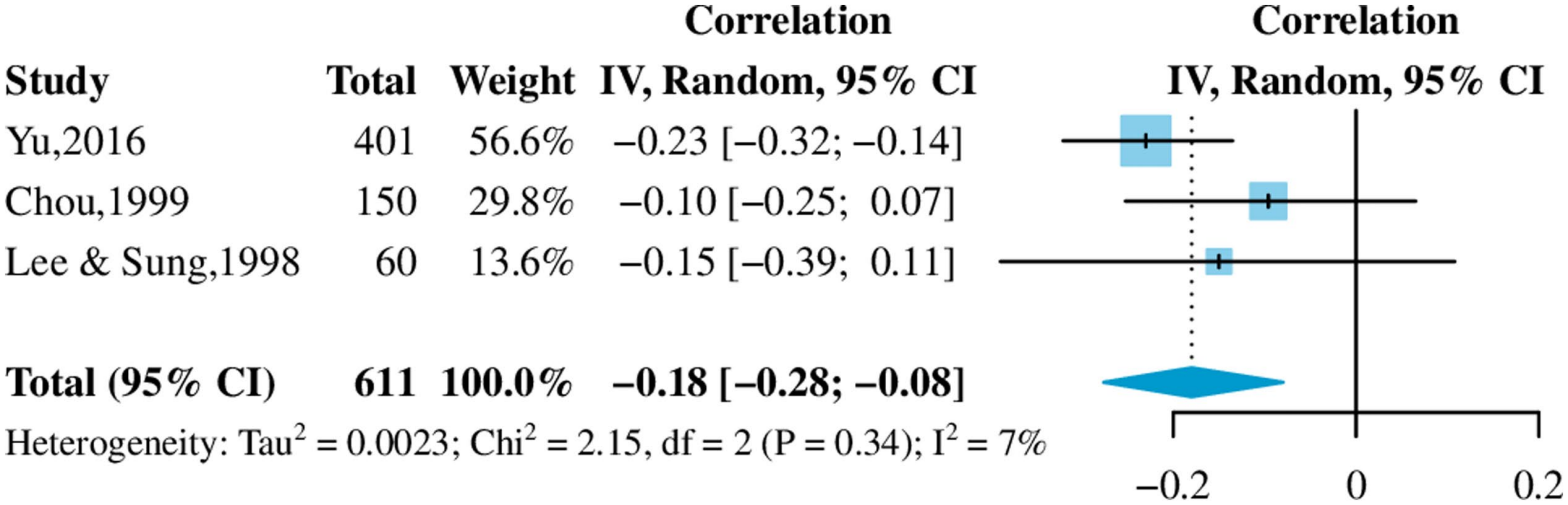
Effect of filial piety on caregiver burden.

Initially, four studies were included in the meta-analysis, but a high between-study heterogeneity was observed (*I^2^* = 86.1%, *p* < 0.001). However, heterogeneity was diminished (*I^2^* = 7.3%, *p* = 0.34) when we excluded a study with an all-female sample (57). Eventually, three studies were included in the meta-analysis, resulting in a sample of 611 dementia CGs. Filial piety was associated with caregiver burden with a correlation coefficient of −0.18 (95%*CI*: [−0.28, −0.08]; Figure 2). Although Lee’s study was not included in the meta-analysis, it was discussed in the review.

### 3.5. Integration of findings

Thematic synthesis identified four themes in line with the aim of the current review: (1) Recognition and understanding of filial piety as part of cultural identity, (2) Role transitions- from child to CG, (3) Filial piety’s constraints on CGs; (4) CGs’ self-compassion through changing cultural norms of filial practice.

#### 3.5.1. Recognition and understanding of filial piety as part of cultural identity

Several studies highlighted that recognizing and adhering to filial culture among East Asian dementia CGs was common (35, 50, 53, 54, 56). For example, in a study by Koo (50), Chinese Singaporean CGs demonstrated deep filial piety and reciprocity for the following reasons: repayment for their parents’ care, practicing a religious faith, and having a Chinese identity. Furthermore, Zhang (35) noted that being filial is an important part of cultural continuity and acts as a future investment. Zhang expands to add that looking after their parents is a child’s responsibility, and it sets an example for their children, who will do the same in the future. It is not only a virtue but an indicator of a person’s character (35). Family CGs embraced filial culture actively or passively.

#### 3.5.2. Role transitions- from child to CG

Several studies explored the impact of filial culture on the role transition of dementia CGs (35, 50, 53–56). In East Asia, filial piety is a cultural belief in which participants assume the caregiving role when their family member becomes impaired and needs assistance. Also, filial piety is a social norm, rather than one’s willingness. Acceptance of this social norm seems to be the most common reason that participants become family CGs (35, 54, 55) and accept their caregiving role (54). In the cycle of filial piety, the relationship between aging parents and their children changes from “parents protecting children” to “children becoming the guardians of their parents” (50). In some East Asian countries, caring for aging parents has even become a legal obligation emphasizing the moral duties of children. This compulsory responsibility enhances and encourages family CGs to take on a caring role (50, 53, 56) and acts as a coping strategy for accepting the role (54).

#### 3.5.3. Filial piety’s constraints on CGs

The influence of filial piety on caregiver burden was controversial. Results of the meta-analysis suggested filial piety may be a protective factor for caregiver burden (*r* = −0.18). However, the study that was not included in the meta-analysis showed that a stronger sense of filial obligation put pressure on the CGs and increased the burden of caregiving (*r* = 0.26) (57). Besides the caregiving burden, filial piety affected other aspects of caregiving such as delaying the diagnosis of dementia and refusing to seek outside help. People may delay recognizing dementia symptoms in this altered relationship because the aged returning to an increasingly dependent state is considered normal (55). The emphasis on filial obligations discourages family CGs from seeking outside help or using formal services because it can disrupt family harmony or be perceived as non-filial (50, 55). Nursing homes are vital formal services for families with dementia. Filial culture pressures create challenges for family CGs during the decision-making process for nursing home placement. Most CGs equated nursing home placement to non-filial practice (51–53) and would receive pressure and criticism from the clan or extended family (51, 52). Constrained by traditional family values and the practice of filial piety, the placement decision created family disagreement, which oftentimes resulted in damaged family relationships (51, 55).

#### 3.5.4. CGs’ self-compassion through changing cultural norms of filial practice

In East Asia, filial piety is considered the root of all virtues. Research by Lee and Sung (49) showed that Koreans CGs exhibited significantly higher scores on filial obligation than Americans. However, with changes in societal norms, people’s practice of filial piety is changing. For example, in some settings, filial piety has altered from traditional to more material forms, such as buying presents (35), finding the best nursing home, and frequently visiting (52). Furthermore, when talking about senior years, some midlife CGs said they do not want to depend on their children (55), releasing them from and legal or moral obligations seen in the past.

## 4. Discussion

This review demonstrated that filial culture’s impact on dementia CGs in East Asia is nuanced yet demand is extensive. Filial culture permeates the whole process of caregiving, from preparing for the CG role to potentially leaving the role and placing the CRs in a nursing home. Before people enter a CG role, filial culture was found to be beneficial for some (35, 54, 55) but detrimental for others, as they felt forced to accept the cultural role (50, 53, 56). In the practice of caregiving, the impact of filial culture manifested in many ways. The meta-analysis revealed that filial culture could reduce care burden, but a study with an all-female sample showed the opposite result (57), possibly suggesting sex based differences. Under the influence of filial culture, some CGs refused to seek external help (50, 55), which affected the diagnosis and treatment of this disease, and their sense of burden. Some people choose to leave the role by placing CRs with dementia in a nursing home, but the pressures of filial culture prevented them from making this choice quickly (51–53) and many disagreed with their family in the process (51, 55).

Filial piety is the fundamental virtue in Confucianism-influenced societies (58). The internalization of filial piety makes abiding by it a means to an end, rather than merely a tool for achieving certain goals for the benefit of society (59, 60). CGs take on roles, refuse to seek outside help, and delay placing CRs in nursing homes to pursue filial piety. For individuals who have strongly internalized a cultural norm, violating this can be psychologically painful (61). Furthermore, CGs could choose safe behaviors to avoid public condemnation. Further research is required to tease out the significant effects of CGs’ behavior under filial pressure on both carer and patient.

Filial culture had controversial effects on dementia and CGs’ well-being because it was diversly conceptualized. Some studies found filial piety not only correlated with reduced burden and stress among CGs (62), but also with an increased quality of care provided (63). Others indicated that filial piety often involved self-suppression, which positively correlated with personal stress and CG burden (64, 65). To integrate these effects of filial piety, Yeh constructed the Dual Filial Piety Model (DFPM), in which he distinguished two sorts of filial piety: authoritarian filial piety (AFP) and reciprocal filial piety (RFP) (66). AFP centers on obedience to parents’ wishes and family order (67), while RFP focuses on the cycle of attachment and responsibility between parents and children (58). Significantly, RFP can promote prosocial development by cultivating empathy, moral identity, and gratitude, regardless of cultural background (68). It has been postulated that CGs of dementia patients benefit more from an atmosphere of RFP because prosocial behavior promotes physical health and buffers against stress (69, 70).

Most of the quantitative studies and meta-analysis results affirmed a positive effect of filial piety on caregiver burden. However, one study came to the opposite conclusion that filial piety increased caregiving stress. We did not include this study in the model because its sample was different from others: all participants in this Korean study were female (*n* = 98), and nearly half (*n* = 48) were not blood-related (daughter-in-law) to CRs. Other qualitative studies had a range of proportions of males CGs (17–47%). All-female participants led to outlying results probably for two reasons: (1) East Asian culture expects females to take more responsibility for looking after the house (71, 72), rather than females’ own choice, it was more likely that culture forced them to become CGs. (2) Female CGs lack sufficient positive interaction with CRs in their daily lives to promote RFP (73). Son preference remains common in countries from East Asia (74), where daughters grow up with fewer resources from their parents than sons (75, 76). Moreover, daughters-in-laws have never lived with their husbands’ parents before marriage, but they are required to care for their in-laws (75). So as female CGs, they may be with low RFP and high AFP. AFP positively correlates with personal stress and maladaptation (e.g., neurotic personality traits, depression and anxiety) (77). Therefore, female CGs may experience more caregiver burden.

With the urbanization and industrialization of society, the connotations and practices of filial culture have changed considerably over time (78), but carers still support and recognize this value strongly. The review found that change in filial practices reflected that CGs in some settings were beginning to consider their own interests while fulfilling their obligations. Some of these adaptations of filial practices included CGs finding it an equally rewarding choice to send their parents gifts and choose a suitable nursing home where they can then visit them frequently, rather than caring for their parents by themselves (35, 51, 79). These changes reflect a few societal shifts. For instance, modernization theory suggests that modernization would lead cultures away from collectivism and toward individualism, where people are less motivated by norms linked to the collective (80) and more driven by self-interest (81–83). Additionally, findings from this review reflected that CGs’ perceptions of filial piety were vastly different from traditional AFP. Conversely, RFP that focused more on emotional connections was perceived as the new norm that strengthened their filial convictions (52, 53). Despite such transformation in filial attitudes, the act of caregiving itself was still heavily associated with the traditional task of fulfillment that emphasize physical and practical support (62). Overall, the recent adaptions of practicing filial piety do not overly weaken the emotional bond between parents and children, at the same, time allow children to provide practical support to parents and practice their own self-compassion, making them better CGs in turn.

## 5. Recommendations for future research and practice

There are significant gaps in the literature on the experiences and needs of East Asian dementia CGs. This systematic review of studies suggests several directions for future inquiry. Most studies used semi-structured individual interviews to explore family dynamics. These qualitative studies explored CGs’ changing roles, CGs decision-making, and experiences of cohesion and conflicts. Findings provided insight into the stresses and challenges that can work for East Asian dementia CGs and should be considered in the design of culturally specific assessments and interventions in the future. Dyadic or group interviews could also be utilized to interview the couple or the family to also glean further insight into the qualitative impact of spousal and family dynamics. The quantitative studies included in this review used well-established scales to measure filial piety and burden, such as Cicirelli’s 7-item scale and Zarit Caregiver Burden Interview (ZBI), which explored the impact of filial culture on CG burden. However, there is a lack of quantitative research on the cultural backgrounds of dementia CGs, with most studies having small sample sizes and involving few variables. Future research should pay more attention to this.

A systematic review of national dementia guidelines noted that some guidelines discussed culture, but that these recommendations were ambiguous. For example, some guidelines recommend using appropriate assessment tools for people who do not speak the local language, but examples are not provided of appropriate tools. Guidelines recommend that health care professionals consider culture when providing care, but few provide examples of how to do so (84). Filial piety was found to have both positive and negative impacts on CG burden. It is important to identify the parts of filial culture that have a positive impact on CGs to support their own strengths within guidelines. Meanwhile, future guidelines for East Asians need to be more cautious about the negative effects of filial piety, which can include delayed disease diagnosis, delayed help-seeking, and a reluctance for social support. As CGs and patients in East Asia tend to view dementia-related symptoms as part of normal aging, professionals also need to be proactive in detecting and assessing patients’ current and changing levels of cognitive function. At the same time, service providers should be concerned about female CGs adherence to traditional cultural values and the stresses and dilemmas that filial piety culture can place on them.

Cultural transition brought about by social development should not be ignored. New connotations and practices of filial culture are more likely to benefit CGs than traditional ones, creating a sense of self-compassion that also benefits the whole family. Future interventions should harness the benefits while respecting the traditional cultural values of the CGs. The unique experiences of CGs in a filial culture suggest that subsequent research should pay more attention to cultural adaptation when designing interventions for this population.

## Author contributions

QW and QY proposed the research questions and designed the study. XX and BQ supervised the data collection and drafted the manuscript. JZ and DJ were responsible for the statistical design of the study and for carrying out the statistical analysis. PS and AW helped with study design and language editing. All authors contributed to the article and approved the submitted version.

## Funding

This study was supported by National Natural Science Foundation of China (71974170) and Leading Innovative and Entrepreneur Team Introduction Program of Zhejiang (2019R01007).

## Conflict of interest

The authors declare that the research was conducted in the absence of any commercial or financial relationships that could be construed as a potential conflict of interest.

## Publisher’s note

All claims expressed in this article are solely those of the authors and do not necessarily represent those of their affiliated organizations, or those of the publisher, the editors and the reviewers. Any product that may be evaluated in this article, or claim that may be made by its manufacturer, is not guaranteed or endorsed by the publisher.
